# Serum alkaline phosphatase was independently associated with depression in patients with cerebrovascular disease

**DOI:** 10.3389/fpsyt.2023.1184673

**Published:** 2023-07-04

**Authors:** Xi Tao, Chen Yang, Juan He, Qianrong Liu, Siyuan Wu, Wenjing Tang, Jia Wang

**Affiliations:** ^1^Department of Neurological Rehabilitation, Hunan Provincial People’s Hospital, Hunan Normal University, Changsha, Hunan Province, China; ^2^Clinical Research Center for Cerebrovascular Disease Rehabilitation in Hunan Province, Changsha, Hunan Province, China; ^3^Hunan Provincical Key Laboratory of Neurorestoratology, Hunan Normal University, Changsha, Hunan, China; ^4^Department of Neurosurgery, Hunan Provincial People’s Hospital, Hunan Normal University, Changsha, Hunan Province, China; ^5^Department of Rehabilitation, Rehabilitation Hospital of Hunan Province, Changsha, Hunan Province, China; ^6^Department of Scientific Research, Hunan Provincial People’s Hospital, Hunan Normal University, Changsha, Hunan Province, China

**Keywords:** vascular depression, cerebrovascular disease, alkaline phosphatase, biomarkers, association

## Abstract

**Background and purpose:**

Blood markers have important value in the diagnosis of depressive disorders. Serum alkaline phosphatase (ALP) not only predicts stroke recurrence and poor functional prognosis in cerebrovascular disease (CVD) patients but also increases significantly in middle-aged women with depression. Thus, it has not been reported whether serum ALP is associated with the development of depression and/or vascular depression (VDe) in CVD patients.

**Methods:**

This was a cross-sectional study of 353 CVD patients (stroke patients, *n* = 291; cerebral small vessel disease (CSVD) patients, *n* = 62). Baseline demographic information, fasting blood markers (such as blood counts, liver function, kidney function and lipids), and brain CT/MRI scans were collected. CVD patients were divided into non-depression, suspected vascular depression (SVD), and positive vascular depression (PVD) groups according to their Hamilton Rating Scale for Depression (HAMD) scores. Univariate analysis of baseline data, blood markers, and the prevalence of lesions (> 1.5 cm) was performed. Subsequently, the diagnostic performance of the univariate and combined variables for SVD and PVD was analyzed using binary logistic regression. The diagnostic value of the multivariate model for VDe was analyzed by ordinal logistic regression.

**Results:**

(1) Serum ALP (*p* = 0.003) and hypersensitive C-reactive protein (hs-CRP, *p* = 0.001) concentrations increased as HAMD scores increased, and the prevalence of brain atrophy (*p* = 0.016) and lesions in the basal ganglia (*p* = 0.001) and parietal (*p* = 0.001), temporal (*p* = 0.002), and frontal lobes (*p* = 0.003) also increased, whereas the concentrations of hemoglobin (Hb, *p* = 0.003), cholinesterase (ChE, *p* = 0.001), and high-density lipoprotein cholesterol (HDL-C, *p* = 0.005) declined. Among these variables, hs-CRP (*r* = 0.218, *p* < 0.001) had a weak positively association with HAMD scores, and ChE (*r* = −0.226, *p* < 0.001) had a weak negative association. (2) The combination of Hb, hs-CRP, ChE, ALP, and HDL-C improved diagnostic performance for VDe [AUC = 0.775, 95% CI (0.706, 0.844), *p* < 0.001]. (3) Hb (OR = 0.986, *p* = 0.049), ChE (OR = 0.999, *p* = 0.020), ALP (OR = 1.017, *p* = 0.003), and basal ganglia lesions (OR = 2.197, *p* < 0.001) were important factors impacting VDe development. After adjusting for Hb, hs-CRP, ChE, HDL-C, lesions in the above mentioned four locations, sex, age and the prevalence of CSVD and brain atrophy, ALP [OR = 1.016, 95% CI (1.005, 1.027), *p* = 0.004] was independently associated with VDe.

**Conclusion:**

Hb, hs-CRP, ChE, ALP, and HDL-C concentrations are potential blood markers of depression in CVD patients and, when combined, may improve diagnostic performance for VDe. Serum ALP was independently associated with VDe in patients with CVD.

## 1. Introduction

Early vascular depression (VDe) is depressive behavior secondary to cerebrovascular disease (CVD) or related to myocardial infarction. Although it has long been considered a type of depressive disorder and has been studied for more than 20 years, it is not included in the 5th edition of the Diagnostic and Statistical Manual of Mental Disorders (DSM-5) (1, 2). Based on etiological and pathological considerations, the diagnosis of VDe is limited to cerebrovascular lesions or the substantial impairment of vascular neurons caused by important vascular risk factors (3). Recently, the academic community has reached a consensus on the core clinical features (e.g., executive dysfunction) of VDe in elderly people (≥ 65 years of age), with diagnostic paradigms that differ from those characteristic of general depression (3). In addition to the criteria of clinical symptomatology, MRI-defined VDe is the second most widely accepted criterion that is required for the diagnosis of depression associated with cerebral small vessel disease (CSVD) (4). However, MRI is limited in its ability to detect VDe because, despite capturing white matter hyperintensities, it overlooks other features of small vessel disease. Compared with that of VDe, the concept of poststroke depression (PSD) is clearer, and the relevant research results for PSD are significantly more informative than those for VDe (5). However, stroke events are rarely independent of CSVD in older patients. Thus, the inclusion of PSD in studies that better characterize VDe as a widespread vasculopathy may contribute to new insights from a variety of perspectives.

In addition to clinical features and elements defined by MRI, blood markers may also contribute to the early diagnosis or prediction of VDe (6). Serum alkaline phosphatase (ALP) is a clinically important indicator of liver function and bone metabolism (7, 8). ALP is derived from multiple different tissues and is tissue-specifically expressed in germ cells, the placenta and intestines. ALP exhibits tissue-nonspecific expression in the brain, liver, kidney and bone; therefore, it is also called tissue-nonspecific alkaline phosphatase (TNAP). Studies have shown that serum ALP is significantly elevated in premenopausal women with depression (8, 9). A recent study of 17,561 adults found significantly higher blood levels of ALP in depressed patients than in controls (10). Similarly, elevated levels of ALP were observed in ovariectomized depressive rats (11). Furthermore, ALP is also an independent predictor of recurrent stroke, adverse functional outcomes, and all-cause mortality in cerebrovascular disease patients (12, 13). However, it has not been reported whether serum ALP concentrations are associated with depression in CVD patients. In this cross-sectional study, we explored the association between serum ALP and VDe in patients with CVD.

## 2. Materials and methods

### 2.1. Study participants

**Figure 1:**
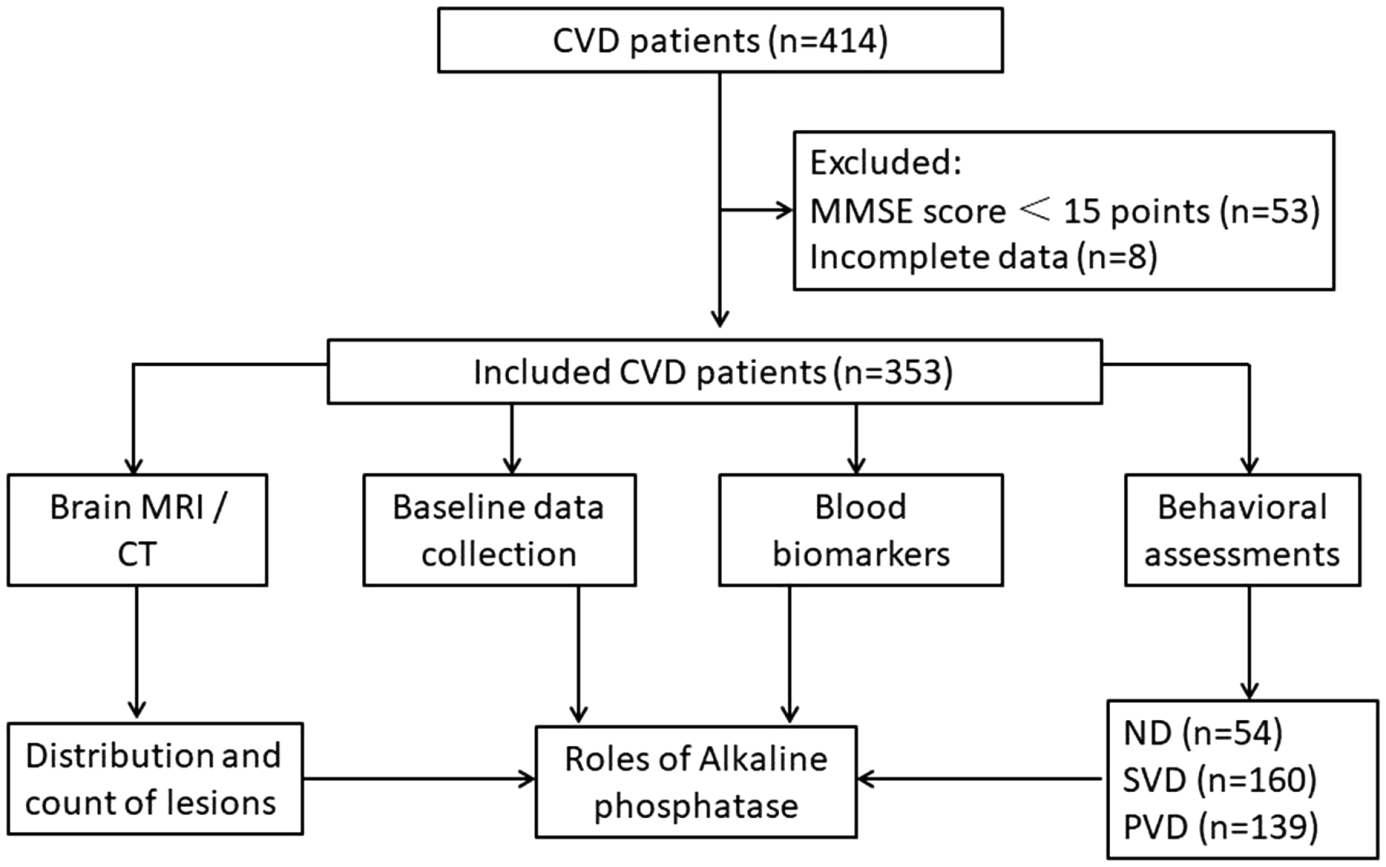
Flow chart of the study. Patients with cerebrovascular disease were recruited (*n* = 414). Among them, patients with an MMSE score less than 15 points (*n* = 53) and incomplete data (*n* = 8) were excluded, and the remaining patients were included in the final analysis (*n* = 353). Demographic data, CT/MRI images, blood markers and some related behavioral scores were collected. Then, a diagnostic model of relevant markers for VDe was constructed.

From May 2020 to July 2021, we collected data from a total of 414 inpatients with CVD from the neurological and neurological rehabilitation departments in a single center. Of these patients, eight with incomplete data and 53 with severe cognitive impairment were excluded. Ultimately, 353 patients were enrolled, including 291 stroke patients and 62 CSVD patients (Figure 1).

The diagnostic requirements or criteria for CSVD or stroke in all patients were based on previously published studies (14–16).

The inclusion criteria were as follows: (1) clear consciousness, (2) a diagnosis of CSVD or unilateral hemispheric stroke, (3) stable vital signs, (4) a Mini Mental State Examination (MMSE) score greater than 15 points, (5) the ability to cooperate to complete behavioral questionnaires, and (6) depressive behavioral symptoms that were relatively stable and had persisted for more than 2 weeks. The exclusion criteria were as follows: (1) infection within 2 weeks before assessment, (2) severe aphasia, (3) suspected history of depression prior to stroke, (4) severe impairment of kidney [a glomerular filtration rate of less than 30 mL/(min × 1.73 m^2^)] or liver (glutamic-pyruvic transaminase concentration of greater than 200 U/L) function, (5) use of antidepressants or benzodiazepines since the onset of depression, and (6) incomplete data.

### 2.2. Clinical features

Baseline demographic information on all possible associations with depression status, including sex, age, educational background, job status, body mass index (BMI), CSVD prevalence, stroke classification and course, previous stroke, coronary heart disease, diabetes, smoking, alcohol consumption, and the presence of gastrointestinal or respiratory nonacute disease, was collected. Of these variables, a history of previous stroke was indexed to the occurrence of a stroke event and hospitalization with or without sequelae. Digestive or respiratory disease referred to chronic diseases (e.g., chronic gastritis, reflux esophagitis, chronic bronchitis, sleep apnea syndrome and stable chronic obstructive pulmonary disease) rather than acute illnesses requiring immediate treatment. Job status (employment) was determined by whether work was performed and the stability of the work.

### 2.3. Behavioral assessments

Patients were assessed for cognitive function and then for depression and anxiety. All behavioral assessments were performed by a senior resident with clinical experience.

The cognitive-behavioral assessment was based on guidelines from the Vascular Impairment Of Cognition Classification Consensus Study (17) and the Chinese framework for the diagnosis and treatment of vascular cognitive impairment (14). Patients’ overall cognitive function was assessed with the MMSE (18).

Low mood, lack of interest, a sense of unworthiness, and psychomotor retardation are the core symptoms commonly associated with depression (19). The diagnostic requirements and exclusion criteria for core depression symptomatology were based on the DSM-5 (19), the “Vascular depression consensus report – a critical update” (3) and the guidelines for the secondary prevention and treatment of depressive impairment in China (20). We then used the 24-item version of the Hamilton Rating Scale for Depression (HAMD) to assess the severity of depression, taking into account the reliability, validity, and breadth of use of the scale in clinical practice (21, 22). The scoring and classification criteria were as follows: (1) HAMD score < 8 points, no depression (normal); (2) eight points ≤ HAMD score < 20 points, suspected depression; (3) twenty points ≤ HAMD score < 35 points, positive depression; and (4) HAMD score ≥ 35 points, major depression. Based on this classification, CVD patients without depression were assigned to a non-depression (ND) group, whereas those with suspected depression were assigned to a suspected vascular depression (SVD) group. Finally, because the number of patients with major depression was only 11, we combined all patients with major depression and patients with positive depression into a single group, which we termed the positive vascular depression (PVD) group. We also used the Montgomery Depression Rating Scale (MDRS) for comparison (23). Additionally, all patients were assessed for anxiety with the Hamilton Rating Scale for Anxiety (HAMA), and their ability to perform activities of daily living was assessed with the modified Barthel Index (mBI) (24).

### 2.4. Blood marker detection

All patients fasted for 8 h, and then their fasting blood was collected (6:00–7:00 in the morning). EDTA anticoagulant was used in the 2 mL sample collected for whole blood analysis (XN-10, JAPAN), which included erythrocyte and lymphocyte counts and hemoglobin (Hb) concentrations. A 5 mL blood sample containing procoagulant was applied for quantitative measurements of biomarkers (HITACHI 7600, JAPAN), including ALP, hypersensitive C-reactive protein (hs-CRP), retinol-binding protein, uric acid, β_2_-microglobulin (β_2_-M), cysteine protease inhibitor C (Cys-C), cholinesterase (ChE), homocysteine (Hcy), triglyceride, lipoprotein α (LP-α), high-density lipoprotein cholesterol (HDL-C), very low-density lipoprotein cholesterol (VLDL-C), low-density lipoprotein cholesterol (LDL-C), and transthyretin concentrations. Commercial kits were used, and certified personnel performed the above analyses. The quantitative measurements were performed using the following methods: the immunoturbidimetric method for hs-CRP, β_2_-M, Cys-C, retinol-binding protein, LP-α and transthyretin; the uricase method for uric acid; the enzyme cycling method for Hcy; the butyryl glucosinolates choline substrate method for ChE; the antibody blockade method for HDL-C; direct method-surfactant clearance method for LDL-C; GPO-PAP method for triglyceride; and NPP substrate-AMP buffer method for ALP.

### 2.5. MRI/CT scanning and analysis

Brain MRI (1.5 T, A Tim System, Germany) imaging data consisted of T1-weighted, T2-weighted, and T2-weighted fluid-attenuated inversion recovery sequences. The thickness was set to 5 mm, and the distance was set to 1.5 mm. Brain CT (64-row, GE Lightspeed spiral CT scanner) was performed as a nonenhanced scan with a thickness and distance of 5 mm. According to the extent of lesion involvement, two experienced senior neurologists manually counted lesions in stroke patients (> 1.5 cm in diameter). Controversial issues were discussed.

### 2.6. Statistical analysis

All data were analyzed using SPSS statistical software version 24.0. (1) Continuous variables, if normally distributed, are shown as the mean ± standard deviation (SD); otherwise, they are expressed as medians (interquartile range, IQR). (2) If all three groups had normal distributions, one-way ANOVA was performed, followed by Tukey’s test or Dunnett’s T3 test as a *post hoc* test. If at least one of the data points among patients with different HAMD scores was not normally distributed, nonparametric and Kruskal–Wallis *H* tests were used, followed by Bonferroni correction for *post hoc* comparisons. (3) The relationships between potential blood markers and behavioral scores were analyzed using the Spearman method based on the distributional characteristics of nonnormal data. An *r* ≤ 0.19 indicated a negligible correlation, 0.20 ≤ *r* < 0.39 indicated a weak correlation, 0.40 ≤ *r* < 0.59 indicated a moderate correlation, 0.60 ≤ *r* < 0.79 indicated a strong correlation, and *r* ≥ 0.80 indicated a very strong correlation (25). (4) The chi-square test was applied to analyze count data. (5) In the absence of depressive disorder as a control, the diagnostic power of univariate and combined variables for PVD or SVD was analyzed with binary logistic regression. (6) Frontal and temporal lobe lesions and other classified variables were used as factors, and continuous variables such as Hb and ALP concentrations were used as covariates. The diagnostic utility of these variables for the various degrees of VDe was analyzed with ordinal logistic regression.

## 3. Results

### 3.1. Comparison of demographic information among patients with different HAMD scores

A total of 353 patients were recruited, 62 with CSVD and 291 with stroke. Nine patients had a history of previous stroke. The full sample was divided into the ND (*n* = 54), SVD (*n* = 160) and PVD (*n* = 139) groups according to the aforementioned HAMD score criteria. The prevalence of CSVD decreased with increasing HAMD scores, with a lower prevalence in the SVD and PVD groups than in the ND group (*χ*^2^ = 15.984, *p* < 0.001). There were no differences in sex, age, employment status, educational background, stroke type, stroke duration, previous stroke, gastrointestinal disorders, respiratory diseases, diabetes, coronary heart disease, smoking, or alcohol consumption among the three groups (Table 1).

**Table 1:** Baseline demographic data among patients with different HAMD scores in the three groups.

Variables	ND (*n* = 54)	SVD (*n* = 160)	PVD (*n* = 139)	*χ^2^/F/H*	*P*
Age (years)^a^	61.19 ± 11.93	60.09 ± 12.75	62.27 ± 13.24	1.081	0.340
Body mass index (kg/m^2^)^b^	24.13 (3.97)	24.17 (3.78)	23.15 (3.95)	6.461	0.040^*^
Sex (male), *n* (%)	37 (68.52)	118 (73.75)	94 (67.63)	1.467	0.480
Education (years), *n* (%)					
0	1 (1.85)	1 (0.63)	6 (4.32)	6.864	0.143
<6	10 (18.52)	21 (13.13)	25 (17.99)		
≥7	43 (79.63)	138 (86.25)	108 (77.70)		
Stroke					
Hemorrhagic stroke, *n* (%)	10 (18.52)	34 (21.25)	43 (30.94)	2.449	0.294
Ischemic stroke, *n* (%)	26 (48.15)	100 (62.50)	83 (59.71)		
Disease duration (months)^b^	3.00 (11.5)	1.20 (3.35)	1.64 (3.33)	5.339	0.069
CSVD, *n* (%)	19 (35.19)	28 (17.50)	15 (10.79)	15.984	0.000^***^
History of stroke, *n* (%)	1 (1.85)	4 (2.50)	4 (2.88)	0.176	0.916
Hypertension, *n* (%)	45 (83.33)	124 (77.50)	113 (81.29)	1.138	0.566
Diabetes mellitus, *n* (%)	20 (37.04)	59 (36.88)	43 (30.94)	1.333	0.513
CHD, *n* (%)	11 (20.37)	34 (21.25)	36 (25.90)	1.149	0.563
Digestive tract disease, *n* (%)	2 (3.70)	8 (5.00)	10 (7.19)	1.141	0.565
Respiratory system disease, *n* (%)	2 (3.70)	7 (4.38)	9 (6.47)	0.924	0.630
Smoking, *n* (%)	15 (27.78)	65 (40.63)	45 (32.37)	3.838	0.147
Alcohol intake, *n* (%)	10 (18.52)	34 (21.25)	27 (19.42)	0.255	0.880
Employment, *n* (%)					
Retired	27 (50.00)	65 (40.63)	64 (46.04)	6.518	0.368
Unemployed	1 (1.85)	6 (3.75)	1 (0.72)		
Liberal profession	7 (12.96)	33 (20.63)	31 (22.30)		
Stable profession	19 (35.19)	56 (35.00)	44 (31.65)		

^*^p <0.05, ^**^ *p* <0.01, ^***^ *p* <0.001.

a Expressed as the mean ± SD.

b Expressed as the median (IQR).

### 3.2. Comparison of blood markers among patients with different HAMD scores

**Figure 2:**
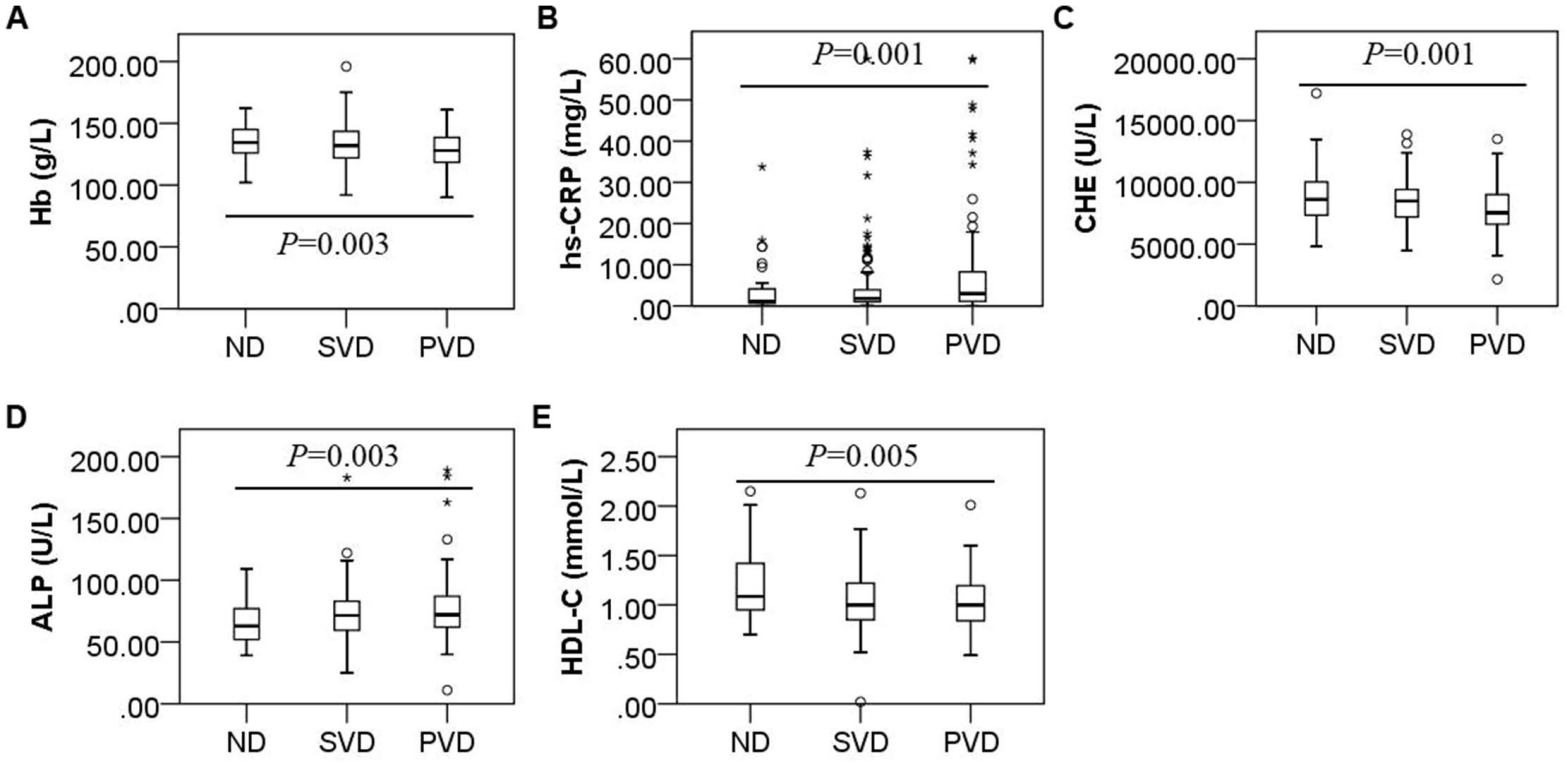
Box plot for the comparison of Hb, hs-CRP, ChE, ALP and HDL-C concentrations among patients with different HAMD scores. Note: **(A)**, **(B)**, **(C)**, **(D)**, and **(E)** represent differences in the concentrations of Hb (*F* = 5.762, *p* = 0.003), hs-CRP (*F* = 15.115, *p* = 0.001), ChE (*F* = 14.656, *p* = 0.001), ALP (*F* = 11.707, *p* = 0.003), and HDL-C (*F* = 10.748, *p* = 0.005) in the non-depression, suspected vascular depression, and positive vascular depression groups, respectively.

Overall, comparisons among the three groups showed that the hs-CRP (*H* = 15.115, *p* = 0.001) and ALP concentrations (*H* = 11.707, *p* = 0.003) gradually increased with increasing HAMD scores, while the Hb (*F* = 5.762, *p* = 0.003), ChE (*H* = 14.656, *p* = 0.001) and HDL-C (*H* = 10.748, *p* = 0.005) concentrations decreased (Figure 2). HDL-C concentrations (*z* = 2.890, *p* = 0.012) decreased, and ALP concentrations (*z* = −2.581, *p* = 0.030) increased in the SVD group; Hb (*t* = 2.698, *p* = 0.020), ChE (*z* = 3.073, *p* = 0.006), and HDL-C (*z* = 3.173, *p* = 0.005) concentrations decreased, and hs-CRP (*z* = −3.652, *p* = 0.001) and ALP (*z* = −3.420, *p* = 0.002) concentrations increased in the PVD group compared with the ND group. Hb (*t* = 2.943, *p* = 0.010) and ChE (*z* = 3.294, *p* = 0.003) concentrations were also obviously reduced in the PVD group compared with the SVD group, whereas hs-CRP concentrations (*z* = −2.643, *p* = 0.025) continued to increase (Table 2).

**Table 2:** Comparison of biomarkers and behavioral scores among the three groups.

Variables	ND (*n* = 54)	SVD (*n* = 160)	PVD (*n* = 139)	*F/H*	*P*	Tukey test with Bonferroni correction		
						ND vs. SVD	ND vs. PVD	SVD vs. PVD
Erythrocytes (× 10^12^/L)^a^	4.44 (0.67)	4.28 (0.71)	4.19 (0.71)	0.088	0.088	/	/	/
Hb (g/L)^b^	134.33 ± 13.23	132.90 ± 15.98	127.53 ± 16.29	5.762	0.003^**^	0.831	0.020^*^	0.010^*^
Lymphocytes (×10^9^/L)^a^	1.79 (0.69)	1.61 (0.83)	1.55 (0.75)	4.724	0.094	/	/	/
hs-CRP (mg/L)^a^	1.12 (3.54)	1.81 (2.92)	3.00 (7.32)	15.115	0.001^**^	0.229	0.001^**^	0.025^*^
Hcy (μmol/L)^a^	14.52 (6.68)	14.75 (6.92)	13.97 (5.37)	1.316	0.518	/	/	/
Retinol-binding protein (mg/L)^a^	37.60 (14.28)	39.20 (12.30)	36.60 (14.30)	3.413	0.181	/	/	/
Uric acid (μmol/L)^a^	343.00 (139.75)	322.50 (135.75)	322.00 (113.00)	3.571	0.168	/	/	/
β_2_-Microglobulin (mg/L)^a^	2.05 (0.72)	2.20 (0.86)	2.10 (0.86)	1.515	0.469	/	/	/
Cystatin C (mg/L)^a^	1.08 (0.36)	1.07 (0.28)	1.08 (0.28)	2.657	0.265	/	/	/
ChE (U/L)^a^	8613.00 (2713.50)	8494.50 (2223.75)	7535.00 (2431.00)	14.656	0.001^**^	1.000	0.006^**^	0.003^**^
ALP (U/L)^a^	63.00 (25.50)	71.50 (23.75)	72.00 (25.00)	11.707	0.003^**^	0.030^*^	0.002^**^	0.660
Triglyceride (mmol/L)^a^	1.58 (1.09)	1.40 (0.81)	1.33 (0.95)	1.613	0.447	/	/	/
HDL-C (mmol/L)^a^	1.09 (0.47)	1.00 (0.37)	1.00 (0.36)	10.748	0.005^**^	0.012^*^	0.005^**^	1.000
LDL-C (mmol/L)^a^	2.68 (1.44)	2.27 (1.32)	2.26 (1.03)	4.933	0.085	/	/	/
LP-α (mg/L)^a^	113.84 (208.78)	170.52 (231.44)	157.83 (251.16)	4.468	0.107	/	/	/
Transthyretin (mg/L)^a^	258.20 (88.78)	270.80 (72.15)	256.20 (85.00)	3.149	0.207	/	/	/
MMSE^a^	28.00 (4.25)	27.50 (4.00)	24.00 (7.00)	42.604	0.000^***^	1.000	0.000^***^	0.000^***^
mBI^a^	100.00 (10.00)	87.50 (45.00)	55.00 (45.00)	77.106	0.000^***^	0.000^***^	0.000^***^	0.000^***^
HAMA^a^	4.00 (4.00)	9.00 (4.00)	13.00 (5.00)	146.995	0.000^***^	0.000^***^	0.000^***^	0.000^***^
HAMD^a^	5.00 (3.00)	14.00 (6.00)	25.00 (8.00)	296.848	0.000^***^	0.000^***^	0.000^***^	0.000^***^
MDRS^a^	4.00 (4.00)	10.00 (6.00)	19.00 (7.00)	248.311	0.000^***^	0.000^***^	0.000^***^	0.000^***^

^*^p <0.05, ^**^ *p* <0.01, ^***^ p <0.001.

a Expressed as the median (IQR).

b Expressed as the mean ± SD.

### 3.3. Comparison of behavioral scores among patients with various HAMD scores

All patients completed the HAMA, HAMD, MDRS, mBI, and MMSE instruments. Patients in the SVD group had lower mBI (*z* = 3.969) scores and higher HAMA (*z* = −5.577), HAMD (*z* = −6.667) and MDRS (*z* = −6.244) scores (all *p* < 0.001) than those in the ND group, while those in the PVD group had not only worse anxiety and depression scores but also significantly lower MMSE scores than controls (*z* = 4.945, *p* < 0.001). Compared with the SVD group, patients in the PVD group had decreased MMSE (*z* = 5.842) and mBI (*z* = 5.999) scores (all *p* < 0.001) and elevated HAMD (*z* = −12.643), HAMA (*z* = −8.204), and MDRS (*z* = −11.455) scores (all *p* < 0.001) (Table 2).

### 3.4. Association between blood markers and behavioral scores

We analyzed associations of potential diagnostic value between blood markers and behavioral scores (n = 353). We found that Hb concentrations were positively correlated with MMSE (*r* = 0.239) and mBI (*r* = 0.182) scores (all *p* < 0.01) and negatively correlated with HAMA (*r* = −0.130), HAMD (*r* = −0.185), and MDRS (*r* = −0.200) scores (all *p* < 0.05). hs-CRP concentrations were negatively correlated with MMSE (*r* = −0.228) and mBI (*r* = −0.275) scores (all *p* < 0.001) but positively correlated with HAMA (*r* = 0.112), HAMD (*r* = 0.218) and MDRS (*r* = 0.215) scores (all *p* < 0.05). ChE concentrations were positively correlated with MMSE (*r* = 0.267) and mBI (*r* = 0.187) scores (all *p* < 0.001) but negatively correlated with HAMA (*r* = −0.174), HAMD (*r* = −0.226) and MDRS (*r* = −0.235) scores (all *p* < 0.01). ALP concentrations were negatively correlated with mBI (*r* = −0.269) scores but positively correlated with HAMA (*r* = 0.109), HAMD (*r* = 0.144), and MDRS (*r* = 0.136) scores (all *p* < 0.05). HDL-C concentrations were positively correlated with mBI (*r* = 0.193) scores but negatively correlated with HAMD (*r* = −0.130) and MDRS (*r* = −0.136) scores (all *p* < 0.05). No correlation was found between ALP or HDL-C concentrations and MMSE scores (Table 3).

**Table 3:** Correlation analysis between blood biomarkers and behavioral scores.

Variables		MMSE	mBI	HAMA	HAMD	MDRS
Hb	*r*	0.239	0.182	−0.130	−0.185	−0.200
	*P*	0.000^***^	0.001^**^	0.014^*^	0.000^***^	0.000^***^
hs-CRP	*r*	−0.228	−0.275	0.112	0.218	0.215
	*P*	0.000^***^	0.000^***^	0.036^*^	0.000^***^	0.000^***^
ChE	*r*	0.267	0.187	−0.174	−0.226	−0.235
	*P*	0.000^***^	0.000^***^	0.001^**^	0.000^***^	0.000^***^
ALP	*r*	−0.099	−0.269	0.109	0.144	0.136
	*P*	0.064	0.000	0.040	0.007	0.011
HDL-C	*r*	0.046	0.193	−0.063	−0.130	−0.136
	*P*	0.390	0.000	0.241	0.014	0.010

^*^p <0.05, ^**^ *p* <0.01, ^***^ *p* <0.001.

### 3.5. Comparison of brain atrophy and lesion counts among patients with different HAMD scores

We categorized and counted all lesions with a diameter greater than 1.5 cm and brain atrophy based on CT or MRI scans of the brain. The results showed that with the increase in HAMD scores, the prevalence of brain atrophy (*χ*^2^ = 8.286, *p* = 0.016) and lesions in the parietal (*χ*^2^ = 14.966, *p* = 0.001), frontal (*χ*^2^ = 11.731, *p* = 0.003), and temporal (*χ*^2^ = 12.283, *p* = 0.002) lobes and basal ganglia (*χ*^2^ = 14.835, *p* = 0.001) in the three groups also increased gradually. The proportion of residual lesions counted did not differ among the three groups (Table 4).

**Table 4:** Comparison of lesion sites among the three groups.

Variables^a^	ND (*n* = 54)	SVD (*n* = 160)	PVD (*n* = 139)	*Χ^2^*	*P*
Brain atrophy	38 (70.37)	90 (56.25)	99 (71.22)	8.286	0.016^*^
Frontal lobe	5 (9.26)	28 (17.50)	41 (29.50)	11.731	0.003^**^
Parietal lobe	1 (1.85)	26 (16.25)	35 (25.18)	14.966	0.001^**^
Temporal lobe	2 (3.70)	25 (15.63)	34 (24.46)	12.283	0.002^**^
Occipital lobe	4 (7.41)	19 (11.88)	12 (8.63)	1.324	0.516
Insular lobe	1 (1.85)	9 (5.63)	12 (8.63)	3.749	0.153
Thalamus	4 (7.41)	15 (9.38)	17 (12.23)	1.204	0.548
Cerebellum	3 (5.56)	9 (5.63)	7 (5.04)	0.055	0.973
Hippocampus	2 (3.70)	2 (1.25)	1 (0.72)	2.026	0.363
Brain stem	9 (16.67)	27 (16.88)	25 (17.99)	0.081	0.960
Basal ganglia	12 (22.22)	52 (32.50)	68 (48.92)	14.835	0.001^**^
Lateral ventricle	3 (5.56)	6 (3.75)	13 (9.35)	4.021	0.134
Corona radiata	5 (9.26)	17 (10.63)	13 (9.35)	0.166	0.921
Centrum ovale	1 (1.85)	6 (3.75)	6 (4.32)	0.773	0.680
Corpus callosum	2 (3.70)	6 (3.75)	8 (5.76)	0.775	0.679
Subtentorial stroke	10 (18.52)	31 (19.38)	26 (18.71)	0.031	0.985

^*^p <0.05, ^**^ *p* <0.01, ^***^ *p* <0.001.

a Indicates *n* %.

### 3.6. Diagnostic performance of combined variables for PVD or SVD

**Figure 3:**
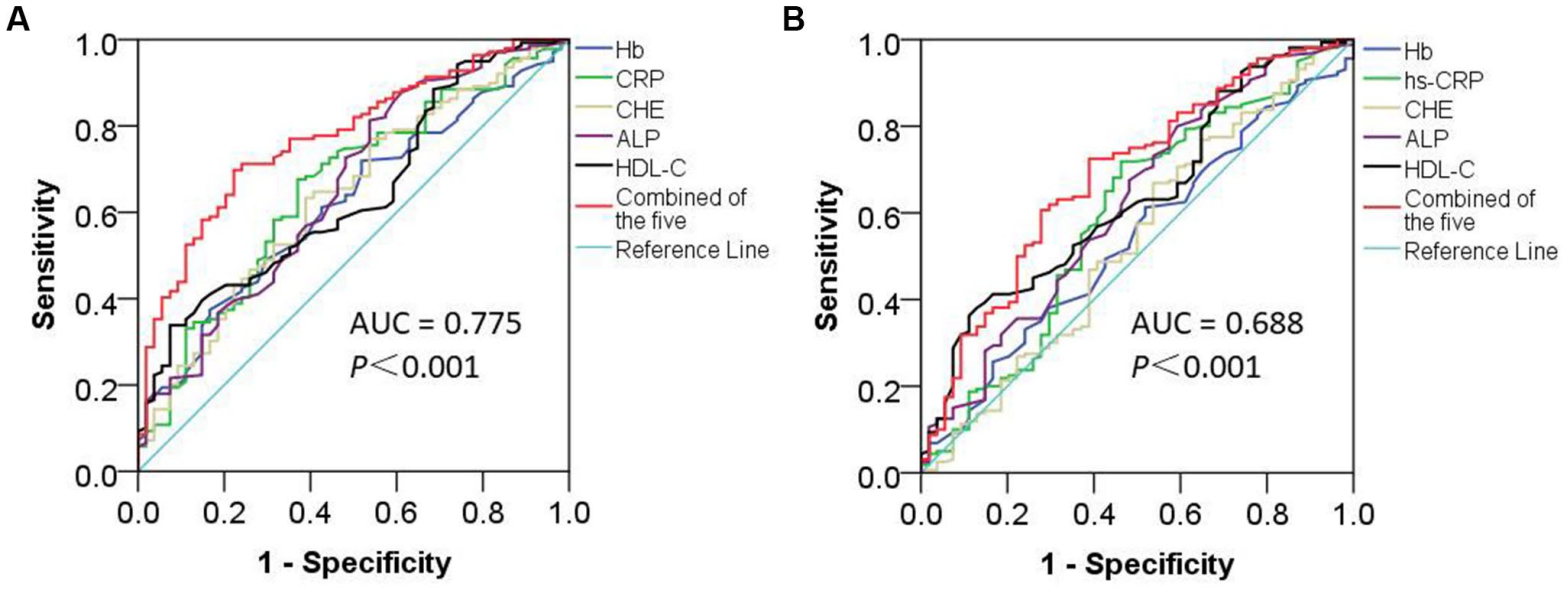
ROC curves of Hb, HDL-C, ChE, ALP, and hs-CRP concentrations and their combination in the diagnosis of PVD and SVD. The AUC of the ROC curves for the combination of Hb, HDL-C, ChE, ALP, and hs-CRP concentrations in the diagnosis of patients with PVD **(A)** was 0.775 [95% CI (0.706, 0.844), *p* < 0.001]. However, in patients with SVD **(B)**, the AUC was 0.688 [95% CI (0.606, 0.771), *p* < 0.001].

For patients with PVD, ROC curves were drawn with Hb, hs-CRP, ChE, ALP and HDL-C concentrations as the independent variables. The results were as follows: (1) Hb concentrations: the area under the curve (AUC) was 0.620 (*p* = 0.009), the 95% confidence interval (CI) was 0.536 to 0.704, the specificity was 83.33%, the sensitivity was 37.41%, and the cutoff value was 122.50 g/L. (2) hs-CRP concentrations: the AUC was 0.656 (*p* = 0.001), the 95% CI was 0.570 to 0.741, the specificity was 62.96%, the sensitivity was 67.63%, and the cutoff value was 1.61 mg/L. (3) ChE concentrations: the AUC was 0.638 (*p* = 0.003), the 95% CI was 0.552 to 0.725, the specificity was 61.11%, the sensitivity was 63.31%, and the cutoff value was 8346.00. U/L. (4) ALP concentrations: the AUC was 0.657 (*p* = 0.001), the 95% CI was 0.569 to 0.745, the specificity was 46.30%, the sensitivity was 81.29%, and the cutoff value was 60.50 U/L. (5) HDL-C concentrations: the AUC was 0.642 (*p* = 0.002), the 95% CI was 0.558 to 0.726, the specificity was 92.59%, the sensitivity was 33.81%, and the cutoff value was 0.875 mmol/L. (6) For the combination of Hb, hs-CRP, ChE, ALP, and HDL-C concentrations, the AUC was 0.775 (*p* < 0.001), the 95% CI was 0.706 to 0.844, the specificity was 77.78%, the sensitivity was 69.78%, and the cutoff value was 0.720. These results suggested that there was a significant improvement in diagnostic performance (Figure 3A).

Similarly, ROC curves were plotted for Hb, hs-CRP, ChE, ALP, and HDL-C concentrations as diagnostic targets for patients with SVD. The results were as follows: (1) For Hb concentrations, the AUC was 0.537 (*p* = 0.416), the 95% CI was 0.450 to 0.624, the specificity was 48.15%, the sensitivity was 61.25%, and the cutoff value was 136.50 g/L. (2) For hs-CRP concentrations, the AUC was 0.593 (*p* = 0.042), the 95% CI was 0.500 to 0.685, the specificity was 53.70%, the sensitivity was 71.88%, and the cutoff value was 1.14 mg/L. (3) For ChE concentrations, the AUC was 0.536 (*p* = 0.434), the 95% CI was 0.442 to 0.629, the specificity was 46.30%, the sensitivity was 66.88%, and the cutoff value was 9124.50 U/L. (4) For ALP concentrations, the AUC was 0.619 (*p* = 0.009), the 95% CI was 0.530 to 0.708, the specificity was 40.74%, the sensitivity was 80.00%, and the cutoff value was 57.50 U/L. (5) For HDL-C concentrations, the AUC was 0.636 (*p* = 0.003), the 95% CI was 0.553 to 0.719, the specificity was 87.03%, the sensitivity was 38.13%, and the cutoff value was 0.915 mmol/L. (6) For the combination of the five markers, the AUC was 0.688 (*p* < 0.001), the 95% CI was 0.606 to 0.771, the specificity was 61.11%, the sensitivity was 72.50%, and the cutoff value was 0.730. These results suggested that there was improved diagnostic performance (Figure 3B).

### 3.7. Multivariate logistic regression analysis to develop a diagnostic model of vascular depression

A VDe diagnostic model (**Model 1**) was constructed with the parietal, frontal, and temporal lobes and basal ganglia as factor variables and Hb, hs-CRP, ChE, ALP, and HDL-C concentrations as covariates. The results showed a good fit (Pearson *χ*^2^ = 653.424, *p* = 0.831). The model fit information was shown to be superior to that of the constant model only (*p* < 0.001). The parallel line test showed that the proportional advantage hypothesis was supported (*χ*^2^ = 14.991, *p* = 0.091). Parameter estimation showed that Hb (OR = 0.986, *p* = 0.049), ChE (OR = 0.999, *p* = 0.020) and ALP (OR = 1.017, *p* = 0.003) concentrations had diagnostic value. The diagnostic accuracy of the combined variables was 55.52%. After adjustment for sex, age and the prevalence of brain atrophy and CSVD, **Model 2** still showed a good fit (Pearson *χ*^2^ = 650.510, *p* = 0.831). The model fitting information showed that it was better than the constant model (*p* < 0.001). The parallel line test showed that the proportional advantage hypothesis was supported (*χ*^2^ = 19.441, *p* = 0.078). Parameter estimates showed that only the ALP concentration had independent diagnostic value (OR = 1.016, *p* = 0.004). In addition, both models showed the importance of basal ganglia lesions (OR = 2.197 ~ 2.219, all *p* < 0.01) in the development of VDe (Table 5).

**Table 5:** Multivariate diagnosis of vascular depression by ordinal logistic regression.

Variables	OR (95% CI)	*P*	^#^Adjusted OR (95% CI)	*P*
Frontal lobe	1.201 (0.622, 2.319)	0.585	1.149 (0.591, 2.232)	0.683
Parietal lobe	1.473 (0.692, 3.135)	0.315	1.513 (0.701, 3.268)	0.292
Temporal lobe	1.606 (0.792, 3.257)	0.189	1.682 (0.819, 3.460)	0.157
Basal ganglia	2.197 (1.418, 3.401)	0.000^***^	2.219 (1.377, 3.571)	0.001^**^
Hb	0.986 (0.973, 1.000)	0.049^*^	0.988 (0.974, 1.002)	0.086
hs-CRP	1.023 (0.994, 1.052)	0.117	1.020 (0.991, 1.050)	0.173
ChE	0.999 (0.999, 1.000)	0.020^*^	0.999 (0.999, 1.000)	0.084
ALP	1.017 (1.006, 1.028)	0.003^**^	1.016 (1.005, 1.027)	0.004^**^
HDL-C	0.625 (0.286, 1.365)	0.238	0.555 (0.244, 1.264)	0.161

^*^p <0.05, ^**^ *p* <0.01, ^***^ *p* <0.001. # indicates adjustment for age, sex, brain atrophy, and CSVD.

## 4. Discussion

The novel findings of our study are as follows. (1) Hb, hs-CRP, ChE, ALP, and HDL-C concentrations are potential diagnostic blood markers in patients with VDe. (2) The proportion of lesions in the parietal, frontal, and temporal lobes and basal ganglia progressively increased with the depressive score. (3) The combination of Hb, hs-CRP, ChE, ALP, and HDL-C concentrations can improve diagnostic performance for VDe. (4) Serum ALP concentrations are independently related to VDe in patients with CVD.

Peripheral biomarkers are important diagnostic adjuncts to disease or functional disability. The selection of economical, reliable, and easy-to-use blood markers not only contributes to the popularization of diagnostic tools but also offers potential ideas for developing new therapeutic approaches that benefit more patients (26). Compared with univariate tests, multivariate tests are useful in elucidating VDe pathogenesis from multiple pathological pathways, thereby improving diagnostic performance.

In our study, Hb, hs-CRP, ChE, ALP and HDL-C concentrations were identified as potential peripheral biomarkers of VDe by univariate analysis. A significant association between anemia and depression was found in a cross-sectional survey of middle-aged and elderly (50 to 75 years) community volunteers (27). In another cohort of young adults, the prevalence of anemia was significantly dose-responsive to depression severity (28). A recent study showed that postpartum anemia increases the risk of postpartum depression (29). This is the first study to show that depression scores increase with decreasing Hb concentrations in patients with CVD. There was also a statistically weak negative correlation. hs-CRP concentrations are a nonspecific marker of inflammation produced by hepatocytes. Plasma concentrations of hs-CRP were significantly higher in patients with PSD than in those without PSD (30). The early elevation of plasma hs-CRP concentrations in patients with ischemic stroke independently predicts the development of PSD 6 months after stroke (31). This study confirms previously reported associations of depressive behavioral symptoms with a nonspecific inflammatory response in patients with CVD (31, 32). The comorbidity of cognitive and depressive symptoms is common. ChE inhibitors have been shown to inhibit the hydrolytic action of ChE, and increased ChE activity has been shown to effectively ameliorate cognitive performance in Alzheimer’s disease patients, but the accompanying behavioral manifestations of depression in these patients have not shown the expected remission rates (33, 34). This study showed that plasma ChE concentrations progressively decreased as depression scores increased. Correlation analysis also supported this weak negative association. Therefore, can ChE concentrations serve as a potential biomarker for VDe diagnosis? Does the provision of ChE help relieve depression? More research is needed to explore this association and its value. In contrast, the potential value of ALP concentrations as a predictor and prognostic agent of acute CVD has been shown in several studies (12, 35, 36), even as a metabolic feature of depression in premenopausal women (9). In the nervous system, TNAP mediates calcification in cerebrovascular smooth muscle cells and neurotransmitter synthesis, and its elevated expression in the hippocampus and serum is associated with decreased cognitive function in patients with Alzheimer’s disease (37, 38). In this study, serum ALP concentrations in CVD patients increased with the depressive score, which has not been previously reported. HDL-C is a major class of lipids with nontraditional functions such as antioxidant, anti-inflammatory, and antithrombotic functions, endothelial cell repair, and microRNA transport (39). Several studies have revealed that lower HDL-C concentrations increase the risk of depression (40, 41). Compared with patients without PSD, the plasma HDL-C of PSD patients decreased significantly (42). Another study of electroconvulsive therapy in specific groups of patients with depression demonstrated, from a lateral perspective, that improvement in depressive behavior is associated with an increase in APOA1, a major component of HDL cholesterol (43). In this study, HDL-C plasma concentrations were reduced in patients with both depression and suspected depression compared with patients without depression, consistent with previous reports (42–44). Based on these studies, Hb, hs-CRP, ChE, ALP, and HDL-C concentrations may play an important role in the occurrence and development of depression in patients with CVD.

Because of its clinical features similar to those of frontal lobe syndrome, VDe was initially thought to be pathogenetically compatible with the anatomical features of “neural circuit dysfunction.” The frontal and prefrontal lobes and their adjacent gyri are the earliest brain regions thought to be involved in emotional disturbances (45, 46). A study using the technology of diffusional kurtosis imaging in neural circuits found that lesions of the microstructure of the subcortical white matter in the temporal and frontal lobes were strongly associated with PSD (47). Another study of depressive behavioral characteristics revealed that frontal lobe lesions in patients with PSD may be associated with higher levels of catastrophic reactions and loss of emotional control, and parietal lobe lesions may increase pain sensitivity (48). Recently, a study based on voxel behavior mapping showed that lesions in the right basal ganglia increased the risk of depression 6 months after stroke (49). Indeed, the neurocircuits associated with depression are not confined to these areas, and the limbic system-cortical-striatal-pallor-thalamic circuitry is widely accepted to be involved in the pathogenesis of mood disorders (50–52). We found that the prevalence of brain atrophy and lesions in the parietal, frontal, and temporal lobes and basal ganglia were different in stroke patients. Specifically, the proportion of lesions increased with the depression score. This suggests that these brain regions are involved in the “disconnection” mechanism of the neural circuitry during the course of VDe, which is consistent with previous reports (47–49).

The academic consensus is that the combination of multiple markers can improve diagnostic performance. In this study, we found that either sensitivity (Hb and HDL-C, less than 40%) or specificity (ALP, less than 45%) was very low for some markers. This reflects the limitations of using a single marker. However, the combination of Hb, hs-CRP, ChE, ALP, and HDL-C concentrations significantly improved the diagnostic performance for PVD (AUC = 0.775) and SVD (AUC = 0.688) in patients with CVD. These findings demonstrate the advantages of combining multiple markers for diagnosis and lay the groundwork for the clinical application of markers that reflect different pathological features in VDe.

Ordinal logistic regression was applied to further analyze the diagnostic meaning of these blood markers for the development of depression in patients with CVD. Based on univariate analysis, we used five blood markers (Hb, hs-CRP, ChE, ALP, and HDL-C concentrations) as covariates, with the temporal, parietal, and frontal lobes and basal ganglia as factors. Initial **Model 1** showed that Hb, ChE, and ALP concentrations were diagnostic of VDe. Then, we adjusted for age, sex and the prevalence of brain atrophy and CSVD. **Model 2** showed that ALP concentrations were an independent diagnostic factor for VDe.

Despite the heterogeneous characteristics of the serum origin of ALP, human and rodent brain tissues express higher levels of TNAP (37, 53, 54). In a mouse model of sepsis, TNAP mediated vascular endothelial cell damage that led to BBB destruction, resulting in decreased interest in spatial exploration and a reduction in spontaneous locomotion (55). Lipopolysaccharide is often used to induce inflammation in rodent models of depression or to validate molecular mechanisms *in vitro* (56). TNAP has been shown to block lipopolysaccharide binding to TLR4 ligands, thereby reducing the inflammatory response (57). In ALP knockout mice, vitamin B6-dependent impairment of homeostasis reduced glutamic acid decarboxylase activity and 5-hydroxytryptamine synthesis (58, 59), which play an important role in the development of depression. Furthermore, membrane TNAP neutralizes the increase in ATP in the extracellular space due to stresses such as ischemia, hypoxia, and hypoglycemia, thereby attenuating the activation of P2X7R and downstream inflammatory pathways in vascular endothelial cells and glial cells (60–62). These findings suggest that TNAP not only has weak anti-inflammatory functions but may also be a molecular basis for structural and functional destruction of the BBB, thus playing an important role in the pathogenesis of VDe. However, the exact molecular mechanism of TNAP needs further validation.

There are some limitations to our research: (1) the sample size of the ND group was small; (2) data on executive dysfunction, reflecting an important feature of cognitive impairment, are lacking in patients with VDe; and (3) there are a limited number of institutions with data sources and a paucity of patients in the community. Future studies, particularly cross-sectional or even longitudinal studies, could focus on patients with different CVDs from multiple centers or community sources based on a given category, thereby further exploring the association between ALP and VDe or its possible value in diagnosing VDe.

## 5. Conclusion

Hb, hs-CRP, ChE, ALP, and HDL-C concentrations are potential blood markers of depression in patients with CVD. The combination of these parameters can improve diagnostic performance for VDe. Serum ALP concentrations were independently associated with VDe in patients with CVD.

## Data availability statement

The original contributions presented in the study are included in the article/supplementary materials, further inquiries can be directed to the corresponding authors.

## Ethics statement

The studies involving human participants were reviewed and approved by the Ethics Committee of the Hunan Provincial People’s Hospital (The First Affiliated Hospital of Hunan Normal University) (Human Ethics No.: 2021–60). The patients/participants provided their written informed consent to participate in this study.

## Author contributions

XT and JW: conceived and designed the study. XT, CY, SW, WT, and JH: performed the study. XT and JW: revised the article for intellectual content. XT: wrote the article. All authors contributed to the article and approved the submitted version.

## Funding

This work was supported by the Clinical Medical Technology Innovation Guidance Project of Hunan Province (2021SK50915), Hunan Provincial Natural Science Foundation (2023JJ30349) and Renshu Foundation of Hunan Provincial People’s Hospital (RS201812) to XT, and Scientific Research Project of Hunan Health Committee (202203102866) to JH.

## Conflict of interest

The authors declare that the research was conducted in the absence of any commercial or financial relationships that could be construed as a potential conflict of interest.

## Publisher’s note

All claims expressed in this article are solely those of the authors and do not necessarily represent those of their affiliated organizations, or those of the publisher, the editors and the reviewers. Any product that may be evaluated in this article, or claim that may be made by its manufacturer, is not guaranteed or endorsed by the publisher.

## Figures and Tables

**Table:** 

ALP	alkaline phosphatase
AUC	area under the ROC curve
ChE	cholinesterase
CSVD	cerebral small vessel disease
CVD	cerebrovascular diseases
HAMA	Hamilton Rating Scale for Anxiety
HAMD	Hamilton Rating Scale for Depression
Hb	hemoglobin
Hcy	homocysteine
HDL-C	high-density lipoprotein cholesterol
hs-CRP	high-sensitivity C-reactive protein
IQR	interquartile range
LDL-C	low-density lipoprotein cholesterol
LP-α	lipoprotein α
mBI	modified Barthel index
MDRS	Montgomery Depression Rating Scale
MMSE	Mini Mental State Examination
MRI	magnetic resonance imaging
ND	non-depression
PSD	poststroke depression
PVD	positive vascular depression
ROC	receiver operating characteristic
SD	standard deviation
SVD	suspected vascular depression
TNAP	tissue-nonspecific alkaline phosphatase
VDe	vascular depression
VLDL-C	very low-density lipoprotein cholesterol
